# The influence of smoothness and speed of stand-to-sit movement on joint kinematics, kinetics, and muscle activation patterns

**DOI:** 10.3389/fnhum.2024.1399179

**Published:** 2024-05-09

**Authors:** Woohyoung Jeon, Xuanliang Neil Dong, Ashley Dalby, Chung-Hyun Goh

**Affiliations:** ^1^Department of Kinesiology, University of Texas at Tyler, Tyler, TX, United States; ^2^Department of Mechanical Engineering, University of Texas at Tyler, Tyler, TX, United States

**Keywords:** stand-to-sit, smoothness, muscle synergy, postural stability, hip joint contribution

## Abstract

**Background:**

Stand-to-sit (StandTS) is an important daily activity widely used in rehabilitation settings to improve strength, postural stability, and mobility. Modifications in movement smoothness and speed significantly influence the kinematics, kinetics, and muscle activation patterns of the movement. Understanding the impact of StandTS speed and smoothness on movement control can provide valuable insights for designing effective and personalized rehabilitation training programs.

**Research question:**

How do the smoothness and speed of StandTS movement affect joint kinematics, kinetics, muscle activation patterns, and postural stability during StandTS?

**Methods:**

Twelve healthy younger adults participated in this study. There were two StandTS conditions. In the reference condition, participants stood in an upright position with their feet positioned shoulder-width apart on the force plate. Upon receiving a visual cue, participants performed StandTS at their preferred speed. In the smooth condition, participants were instructed to perform StandTS as smoothly as possible, aiming to minimize contact pressure on the seat. Lower leg kinetics, kinematics, and coordination patterns of muscle activation during StandTS were measured: (1) angular displacement of the trunk, knee, and hip flexion; (2) knee and hip extensor eccentric work; (3) muscle synergy pattern derived from electromyography (EMG) activity of the leg muscles; and (4) postural sway in the anterior–posterior (A-P), medio-lateral (M-L), and vertical directions.

**Results:**

Compared to the reference condition, the smooth condition demonstrated greater eccentric knee extensor flexion and increased joint work in both the knee and hip joints. Analysis of specific muscle synergy from EMG activity revealed a significant increase in the relative contribution of hip joint muscles during the smooth condition. Additionally, a negative correlation was observed between knee extensor and vertical postural sway, as well as hip extensor work and M-L postural sway.

**Conclusion:**

Smooth StandTS facilitates enhanced knee eccentric control and increased joint work at both the hip and knee joints, along with increased involvement of hip joint muscles to effectively manage falling momentum during StandTS. Furthermore, the increased contributions of knee and hip joint work reduced postural sway in the vertical and M-L directions, respectively. These findings provide valuable insights for the development of targeted StandTS rehabilitation training.

## Introduction

Sit-to-stand (STS) and stand-to-sit (StandTS) are daily functional activities that are commonly used in rehabilitation settings to assess the strength, postural stability, and mobility of older adults (Tiedemann et al., 2008; Jeon et al., 2021b). While StandTS may initially seem like a reverse movement of STS, the necessary force control and associated muscle activation patterns differ fundamentally from STS. In contrast to the predominant role of knee extensor concentric contraction in the uprising phase of STS to generate vertical force (Jeon et al., 2019), StandTS requires a distinctive aspect of motor control for descending balance (Ashford and De Souza, 2000). Particularly, knee extensor eccentric control plays a pivotal role in controlling falling momentum against gravity to ensure a stable and safe landing during StandTS (Pavol and Pai, 2007; Jeon et al., 2023c).

Current existing performance-based STS-StandTS clinical tests, designed to assess fall risk in older adults, predominantly emphasize the rapid “standing up” motion to evaluate lower extremity muscle strength and power. However, these clinical assessments tend to overlook the measurement of “balance control” ability associated with fall risk during the descending phase of sitting down, where knee extensor eccentric control plays a key role in maintaining postural stability. Therefore, there is a clinical need to investigate the characteristics of StandTS movement and its connection with postural stability control.

Modifications in movement smoothness and speed in functional movements such as walking and STS influence the kinematics, kinetics, and muscle activation patterns of the movement. These changes significantly require different types of movement control to maintain postural stability during the execution of the movement (Fukuchi et al., 2019; Wang et al., 2021). For example, engaging in abrupt and rapid movements at the ankle and knee joints induces dynamic changes in joint angular displacement, force production pattern, and the associated muscle activation characteristics originating from each joint, thereby affecting overall movement performance (Hager et al., 2020; Zhang et al., 2021).

Similarly, the smoothness and speed of StandTS movement lead to changes in kinematics, kinetics, and muscle activation patterns, influencing dynamic descending balance control during StandTS (Kralj et al., 1990). Therefore, gaining insight into the impact of speed and smoothness variations in StandTS movement on kinematics, kinetics, and muscle activation patterns holds significant value. This understanding can inform the design of effective and personalized rehabilitation training programs for dynamic descending balance control, particularly beneficial for older adults or patients with knee extensor muscle atrophy, often caused by neurological disorders.

The purpose of this study was to investigate (1) the influence of increased smoothness and reduced (slow) speed in StandTS movements on joint kinematics, kinetics, and muscle activation patterns, and (2) the impact of movement characteristics (smooth and slow) on knee and hip joint works, examining their relationship with postural stability during StandTS. The findings of this study can provide insights into novel directions for developing a new clinical assessment using the StandTS movement.

## Materials and methods

### Type of study

Within-subjects design: All participants took part in both the reference and smooth StandTS conditions, allowing for a direct comparison of StandTS performance within the same individuals.

### Participants

In total, 12 healthy younger adults participated in this study (21.5 ± 2.1 years). Physical activity level (the number of days and hours spent walking and doing physical activities per week) was measured using the International Physical Activity Questionnaire (Craig et al., 2003).

Participants were included in this study if they had a “moderate” or higher physical activity level, which meets the following criteria: (a) 3 or more days of vigorous-intensity activity of at least 20 min per day; (b) 5 or more days of moderate-intensity activity and/or walking of at least 30 min per day; or (c) 5 or more days of any combination of walking, moderate-intensity or vigorous-intensity activities achieving a minimum total physical activity of at least 600 min/week.

Participants were excluded from this study if they had (1) deficits or disorders that could affect balance control; (2) a history of dizziness and imbalance; (3) a history of neurological (e.g., Parkinson’s disease, Alzheimer’s disease, stroke, and visual and/or vestibular impairment), musculoskeletal, or any other systemic disorders; and (4) body mass index (BMI) within the overweight and obesity range (BMI is higher than 25 kg/m^2^; Table 1).

**Table 1:** Anthropometrics characteristics of study participants (male and female).

Characteristics	Male (*n* = 6)	Female (*n* = 6)	*p*-value
Anthropometric			
Age (years)	21.33 ± 1.86	21.66 ± 1.63	0.74
Height (cm)	174.20 ± 7.89	165.48 ± 6.53	0.06
Weight (kg)	74.68 ± 6.97	65.70 ± 5.74	0.04*
BMI (kg/m^2^)	24.56 ± 0.32	23.97 ± 1.19	0.26

*Represents a significant difference between men and women. Values are presented as mean ± standard deviation.

The principal investigator (PI) and his research assistants explained the purpose, research procedures, potential risks, and benefits to the prospective participants. The potential participant was asked to read the informed consent form before being allowed to participate, ensuring that they understood the presented information. PI and his research team members were on hand to oversee each participant during the test and conducted data collection according to the data collection protocol.

All procedures were approved by the Institutional Review Board at the University of Texas at Tyler [(IRB#: 2022–118) and dated 10 April 2022] and were in accordance with the 1975 Declaration of Helsinki. All participants provided written informed consent prior to study participation.

### Data collection

**Figure 1:**
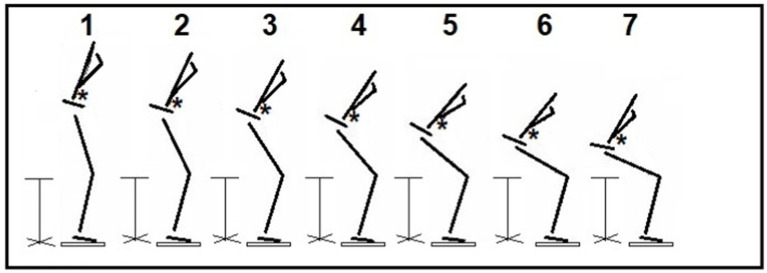
Stand-to-sit (StandTS) movement. This figure illustrates the controlled downward movement during the descending phase of StandTS. The “controlled” falling momentum is well observed during this timeframe. Following the last frame (frame #7), the dropping speed accelerates, quickly increasing vertical ground reaction force, and the subject makes contact with the seat rapidly. The asterisks (*) denote the position of the center of mass (CoM).

For StandTS testing, participants maintained an upright standing position with their feet positioned shoulder-width apart and both feet on a force plate (Bertec Corp., Columbus, OH) in a self-selected parallel foot position. During the StandTS task, participants crossed their arms over their chest and sat down on an armless, backless bench with the back of the knees not touching the bench. The seat height was individually adjusted for each participant based on the distance from the center of the knee joint to the floor, achieving a 90-degree knee angle when seated (Figure 1).

Upon receiving a visual light cue, in the reference condition, participants performed the StandTS task at their preferred speed. In the smooth condition, participants were instructed to perform the StandTS as smoothly as possible, minimizing the contact pressure on the seat. A pressure-sensitive force sensor pad (Smart Caregiver, United States) on the seat was used to determine the initiation point of seating based on the magnitude and distribution of sitting pressure.

In the practice session, participants practiced StandTS once in each condition (reference and smooth). In the test session, three trials were performed for each of the StandTS conditions (reference vs. smooth) in random order. After completing StandTS, participants maintained a sitting position for 5 s. The test lasted approximately 2 h on average and was completed in a single day. All 12 subjects completed the test without any attrition.

#### Kinetics and kinematics

The force plate (Bertec Corp., Columbus, OH) recorded the center of pressure (CoP) and ground reaction force (GRF) on the anterior–posterior (A-P), medio-lateral (M-L), and vertical axes. GRF was normalized to body mass (kg). Measures of the mean velocity of CoP were used to quantify postural sway on the A-P and M-L directions during StandTS (Karlsson and Frykberg, 2000; Jeon et al., 2019, 2021a). The force plate data were collected at 1000 Hz.

The full-body kinematics was recorded using the Vicon motion analysis system (Oxford Metrics Group Ltd., Oxford, United Kingdom). In total, 39 reflective markers were placed on anatomical landmarks based on the full-body modeling (Vicon Nexus 2.12) with Plug-in-Gait (Vicon Motion Systems, Oxford Metrics Group Ltd). Measures of the standard deviation of the center of mass acceleration (SDCoMAccel) of the body were used to quantify postural sway in the vertical direction during StandTS (Jeon et al., 2021a, 2023a). Motion capture data were collected at 100 Hz.

#### Electromyography

A wireless EMG System (BIOPAC Co., United States) was used for the acquisition of muscle activity signals. BIOPAC adhesive pre-gelled Ag/AgCl surface EMG electrodes (size: 11 mm diameter, 35 mm vinyl backing) were placed bilaterally on the tibialis anterior (TA), medial gastrocnemius (mGas), vastus lateralis (VL), biceps femoris (BF), gluteus maximus (Gmax), and gluteus medius (Gmed). The positioning of the electrodes was in accordance with the SENIAM guidelines (Hermens et al., 2000). To normalize the EMG amplitude for each muscle, participants performed maximum voluntary isometric contraction for each muscle before collecting data (Jeon et al., 2021a). EMG data were sampled at 1000 Hz.

### Data processing

All kinetics, kinematics, and EMG variables were computed using a custom-written algorithm in MATLAB (version 2023b, The Mathworks Inc., Natick, MA, United States).

#### Kinetics and kinematics

**Figure 2:**
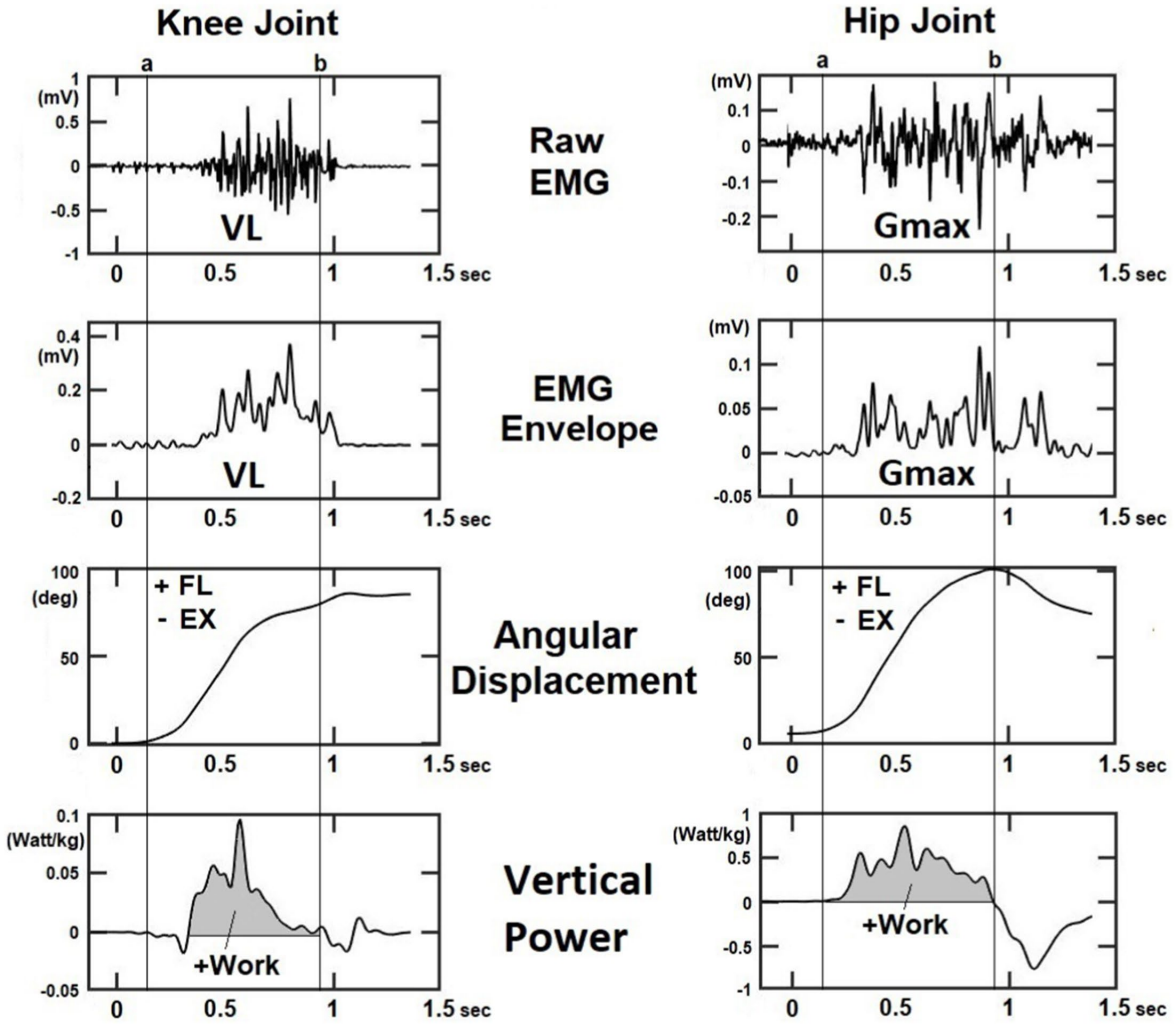
The representative figure illustrates the surface EMG signal after digital smoothing, angular displacement, and power at the knee and hip joints during the StandTS task. Eccentric work was calculated by the integration of the power-time curve, where positive work indicates eccentric activation of the knee extensor (VL) and hip extensor (Gmax) muscles to resist gravitational force and control the descending movement in the vertical direction (shaded area on the vertical power). VL: vastus lateralis; Gmax: gluteus maximus.

The center of mass (CoM) trajectory, joint angular displacement, and power of the body were calculated using Vicon Nexus 2.12 software (Vicon, Oxford Metrics, United Kingdom). The trunk flexion angle was defined as the angle between the thorax and the laboratory coordinate system (the Plug-in Gait model). Kinematic and kinetic data were low-pass filtered through a fourth-order Butterworth filter with a cutoff frequency of 6 Hz and 25 Hz, respectively (Jeon et al., 2021a). Positive eccentric work at the knee and hip joints to resist gravity in the vertical direction during StandTS was calculated by the integration of the power–time curve (Figure 2). All work data were normalized to body weight (J/kg).

#### Electromyography

The raw surface EMG (sEMG) data collected during StandTS underwent the following processing steps:

1. Pre-processing of sEMG signal: Any DC offset was first eliminated using the “detrend” function in MATLAB. Then, a median filter was applied to the signal to remove noise (Feleke et al., 2021), followed by the application of a 20–450-Hz bandpass filter to extract the frequency range where muscular energy is concentrated (Altimari et al., 2012).
2. sEMG rectification and linear envelope: sEMG signal values below zero were converted to positive values of the same amplitude to create a full-wave rectified sEMG signal (see Figure 2). To obtain sEMG envelopes, a second-order Butterworth low-pass filter with a 20-Hz cutoff frequency was applied as a digital smoothing (Jeon et al., 2023c).

#### Muscle synergy

**Figure 3:**
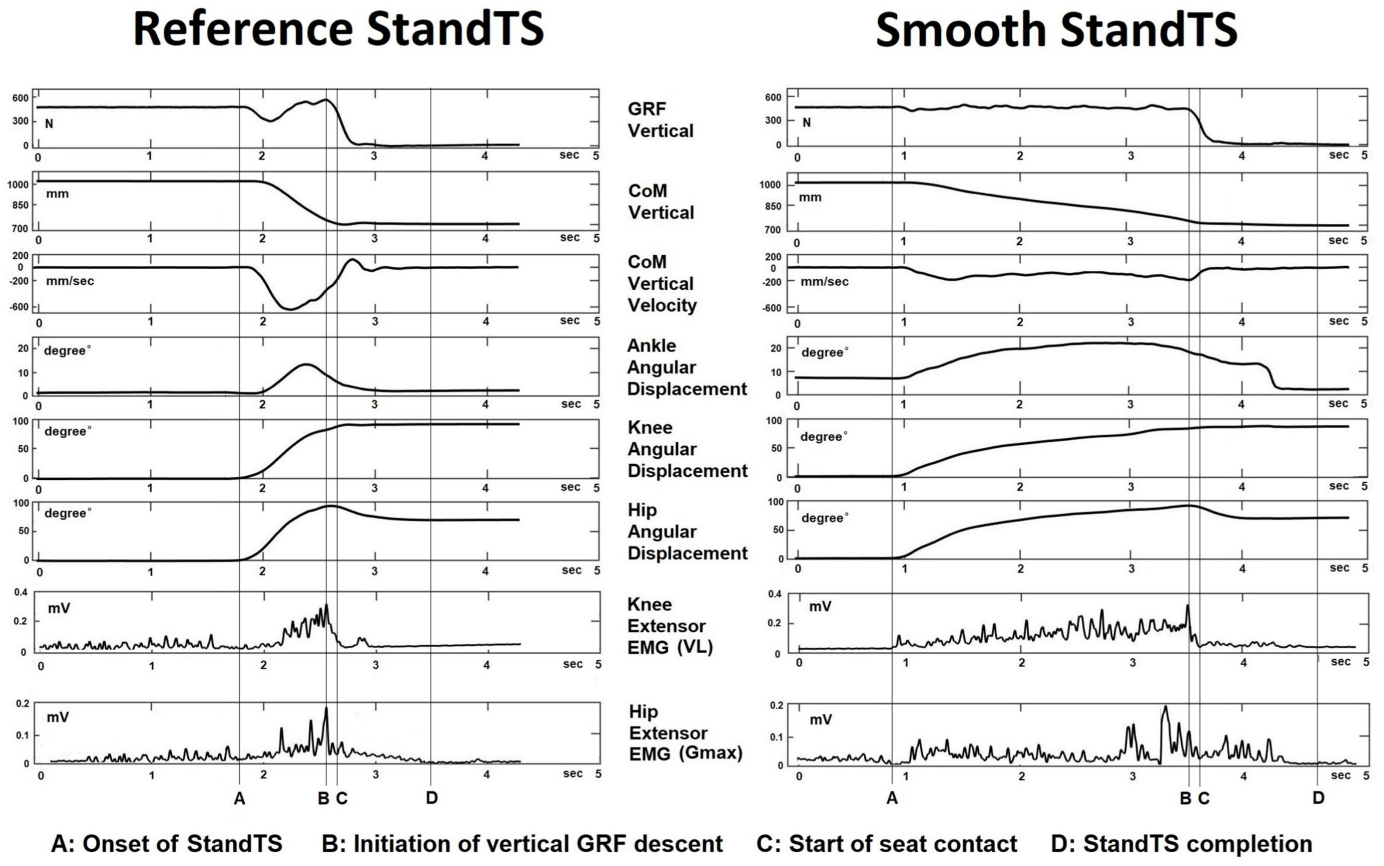
Kinematics, kinetics, and EMG muscle activation at the knee and hip joints during the reference and smooth StandTS conditions from a representative subject.

To characterize the muscle activation patterns for balance recovery strategies, we performed muscle synergy analysis using non-negative matrix factorization (NNMF). Prior to NNMF decomposition for muscle synergy extraction, we employed a moving root mean square (RMS) with a window length of 100 samples to create the sEMG envelopes (Saito et al., 2021). The synergies were extracted from five trials of each StandTS (reference and smooth) condition. An EMG matrix was constructed where the rows are the dominant leg muscles, while the columns are the sampled data during the descending phase of StandTS. The descending phase of StandTS was defined as spanning from the onset of StandTS to just before initiating seat contact (Figures 3A–C).

#### Extraction of muscle synergy

NNMF, using a multiplicative iterative algorithm, extracted muscle synergies (muscle-weighting and temporal synergy activation) from the EMG matrix (Lee and Seung, 1999; Tresch et al., 2006). NNMF decomposes the EMG signals of a specific muscle activation pattern within a given time period (the descending phase of StandTS) into two distinct components:

1. W: This vector specifies the spatial pattern of the relative activation level of each muscle in the muscle synergy. Each muscle’s contribution is relatively weighted within this spatial structure.
2. C: This scaling coefficient represents the temporal synergy activation. The spatial components are multiplied by a scaling (synergy recruitment) coefficient C. This transformation can be expressed as:

(1) $${EMG}_{0}\;\left(m\times t\right)=W\left(m\times n\right)\cdot C\left(n\times t\right)+e=\mathrm{EMGr}\;\left(m\times t\right)+e\text{,}$$

(where *m* = the number of muscles, *t* = the number of time points, *n* = the number of muscle synergies, *e* = residual error, and EMGr = reconstructed EMG matrix)

The spatial components (W) are fixed time-invariant patterns, whereas the temporal synergy activation coefficient (C) varies over time (Torres-Oviedo and Ting, 2007). Therefore, the coefficient (C) specifies how the coordinated muscle activation pattern is modulated over time during the targeted movement period.

To evaluate the similarity between EMG_0_ and EMGr, variability accounted for (VAF) was calculated according to the following equation:


(2) $$VAF=\left(1-\frac{{\left({EMG}_{0}-{EMG}_{r}\right)}^{2}}{{\left({EMG}_{0}-\mathrm{mean}\left({EMG}_{0}\right)\right)}^{2}}\right)\times 100\%$$

To determine the optimal number of synergies, we repeated the optimization to extract k (from 1 to the number of EMG sensors) synergies and the associated VAF. Then, the smallest k with VAF > 90% was selected (Frère and Hug, 2012).

### Statistical analysis

A statistical software (IBM SPSS Statistics 25; Chicago, IL, United States) was used for performing all statistical analyses, with an established *a priori* alpha level of 0.05. For the justification of our sample size, an *a priori* power analysis was conducted using G*Power. Effect size (Cohen’s *d*) was calculated based on previous studies (Jeon et al., 2019, 2021a,b). We detected an effect size of 0.78. Through power calculation, we determined that with 12 participants, there would be 80% power (1 –β) at a 5% level of significance (α). Normality was assessed using the Shapiro–Wilk test.

The mean of three trials of each StandTS condition (reference and smooth) was used for all kinematics, kinetics, and EMG analyses. A one-way ANOVA was used to examine whether the difference between the two StandTS conditions was significantly greater than the variance of the three trials within each StandTS condition.

A one-way repeated-measures ANOVA was used to determine differences in (1) the angular displacement of the knee, hip, and trunk flexion; (2) knee and hip extensor eccentric work; and (3) postural sway (mean velocity of CoP) in the A-P and M-L directions, along with SDCoMAccel in the vertical direction between the two StandTS conditions (reference and smooth). As the vertical postural sway data were not normally distributed, the Mann–Whitney (non-parametric) test was used to compare postural sway in the vertical direction. As the combined postural sway data (reference + smooth) also exhibited a non-normal distribution, Spearman’s correlation (ρ) was conducted to estimate the correlation between joint work and postural sway.

The k-mean clustering algorithm (from MATLAB R2022b) categorized the similar groups of muscle synergies extracted from EMG activity during StandTS across all participants. Subsequently, the intra-class correlation coefficient (ICC) was used to examine the internal consistency of all muscle synergies. Common muscle synergies shared between reference and smooth conditions, with an ICC over 0.75 (indicating good reliability), were categorized in the same cluster. For identifying synergies specific to either the reference or smooth StandTS conditions, muscle synergies with an ICC value over 0.9 (indicating excellent reliability) were categorized in the same cluster (Koo and Li, 2016).

## Results

All data are presented as mean ± standard deviation (SD). The kinematics, kinetics, and muscle synergy data during the descending phase of StandTS were analyzed. Kinetic data were normalized to body mass. For all dependent variables, the difference between two StandTS conditions (reference and smooth) was significantly greater than the variance of the three trials in each StandTS condition (*p* < 0.001).

### Angular displacement of the knee, hip, and trunk during the StandTS descending phase

**Figure 4:**
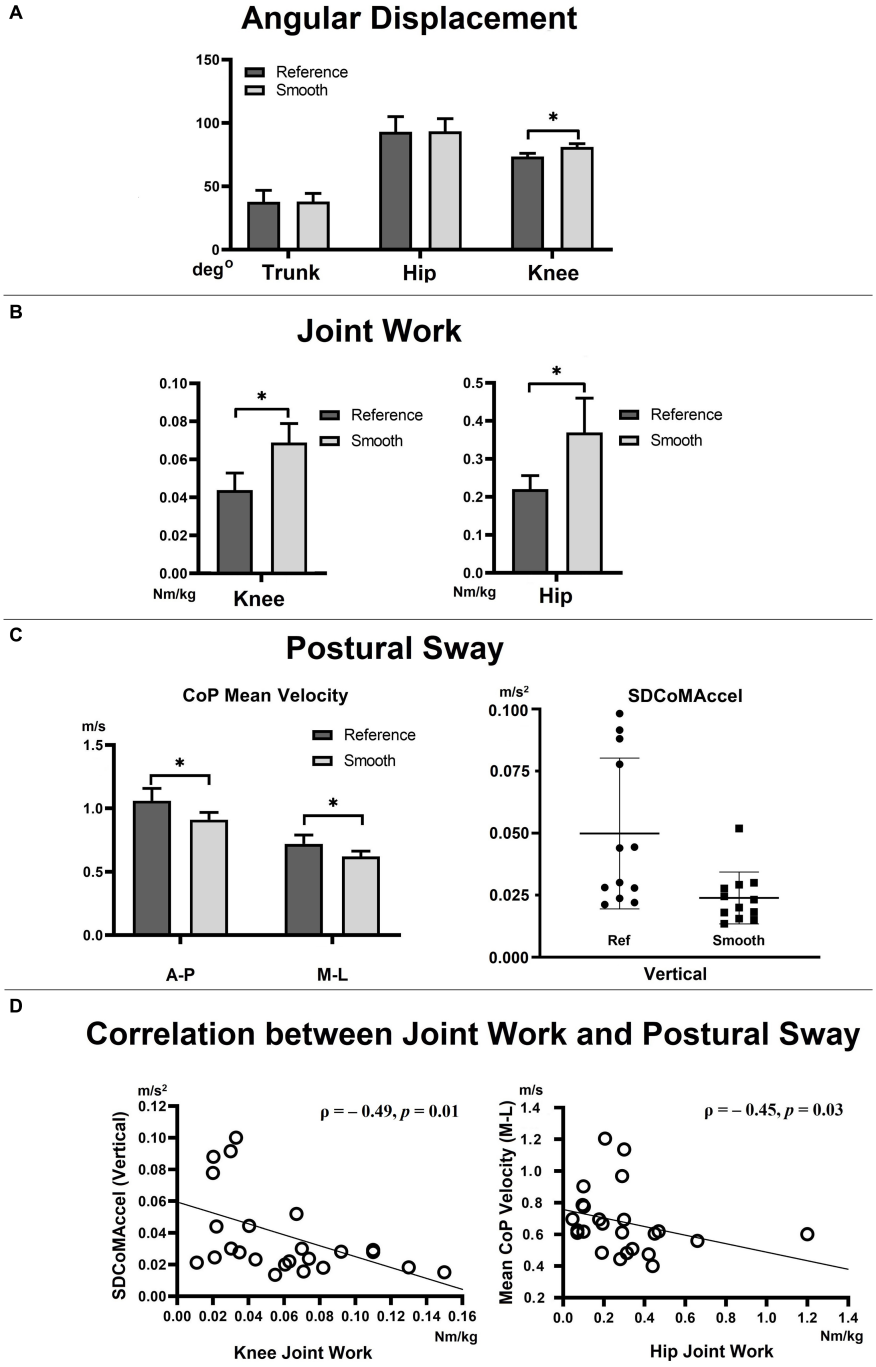
Angular displacement **(A)**, joint work **(B)**, postural sway **(C)**, and correlation between joint work (knee and hip joints) and postural sway **(D)**. CoP: center of pressure, SDCoMAccel: standard deviation of the center of mass acceleration. VL: vastus lateralis; Gmax: gluteus maximus.

There was a main effect of the sitting condition (reference vs. smooth) on knee flexion angular displacement during the StandTS descending control movement. The smooth StandTS demonstrated a greater knee extensor flexion (81.11° ± 8.84°, *p* < 0.01, effect size: η2 = 0.665, observed power = 0.988) than the reference StandTS (73.40° ± 9.26°). However, no significant differences were found in trunk and hip flexion between the two conditions (Figure 4A).

#### Knee and hip joint work

The sitting condition (reference vs. smooth) demonstrated a main effect on joint work at the knee and hip joints. Positive eccentric work, aimed at resisting gravity in the vertical direction during StandTS, was significantly higher in the smooth StandTS in both the knee (0.069 ± 0.01 Nm/kg, *p* < 0.01, effect size: η2 = 0.664, observed power = 0.988) and hip (0.376 ± 0.09 Nm/kg, *p* = 0.02, effect size: η2 = 0.388, observed power = 0.673) joints than the reference StandTS (knee = 0.044 ± 0.009 Nm/kg, hip = 0.216 ± 0.036 Nm/kg) (Figure 4B).

#### Postural sway

In all directions, a significantly reduced postural sway was observed in the smooth StandTS compared to the reference StandTS. The mean velocity of the CoP in the A-P and M-L directions, representing A-P and M-L postural sway, was significantly smaller in the smooth StandTS than the reference StandTS (A-P direction: reference StandTS = 1.06 ± 0.33 m/s, smooth StandTS = 0.91 ± 0.20 m/s, *p* = 0.035, effect size: η2 = 0.345, observed power = 0.593, M-L direction: reference StandTS = 0.72 ± 0.24 m/s, smooth StandTS = 0.62 ± 0.15 m/s, *p* = 0.02, effect size: η2 = 0.403, observed power = 0.700).

In the vertical direction, the SDCoMAccel, representing postural sway in the vertical direction, was also smaller in smooth StandTS (0.02 ± 0.003 m/s^2^, mean rank = 8.67, Mann–Whitney U = 26.00, *p* = 0.01, effect size: η2 = 0.481, and observed power = 0.827) than reference StandTS (0.05 ± 0.01 m/s^2^, mean rank = 16.33) (Figure 4C).

In addition, a negative correlation was found between the SDCoMAccel in the vertical direction (representing vertical postural sway) and eccentric work at the knee joint (ρ = − 0.49, *p* = 0.01), as well as between the mean velocity of the CoP in the M-L direction (representing M-L postural sway) and eccentric work at the hip joint (ρ = − 0.45, *p* = 0.03) (Figure 4D).

#### Muscle synergy

There was no significant difference in the mean value of the number of synergies extracted between the two conditions (reference: 2.5 ± 0.67, smooth: 2.83 ± 0.71).

**Figure 5:**
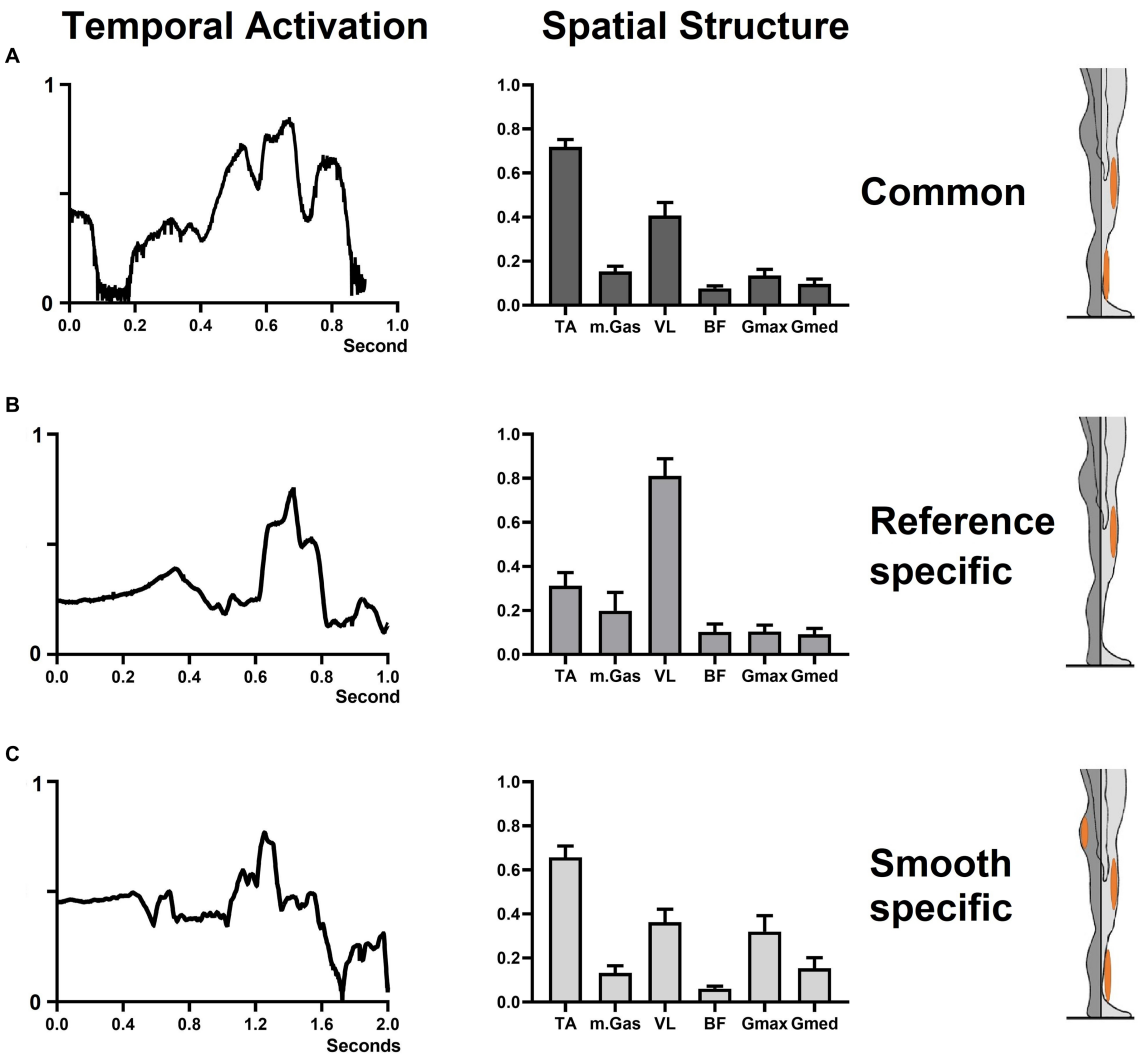
Common and specific muscle synergy during reference and smooth StandTS. The left column displays the temporal recruitment coefficient. The middle column showcases the spatial component, indicating the relative contribution of each muscle. The right column highlights the predominant muscle within the spatial synergy component. **(A)** common muscle synergy; **(B)** reference-specific muscle synergy; **(C)** smooth-specific muscle synergy.

Muscle synergies from all participants were grouped into three clusters (see Figure 5). Within these clusters, we identified both common and specific muscle synergies between the reference and smooth StandTS conditions. We observed a cluster showing good reliability (ICC value ≥0.75) consistently across all participants, which can be determined as a common strategy for both reference and smooth StandTS. This common synergy exhibited predominant activation of TA and VL muscles. Specific muscle synergies displayed excellent reliability (ICC value ≥0.90). We found one specific muscle synergy in the reference StandTS and one specific muscle synergy in the smooth StandTS. The reference-specific muscle synergy cluster had prominent VL muscle activation, while the smooth-specific muscle synergy showed a relatively greater contribution of Gmax and Gmed, along with a similar pattern of TA and VL activation as observed in the common synergy (Figure 5).

## Discussion

### Knee extensor eccentric control during StandTS

Knee extensor eccentric control is important for regulating falling momentum against gravity in the vertical direction, ensuring a stable and safe landing (Jeon et al., 2021b, 2022, 2023c). Knee extensor eccentric control allows for flexibility in adjusting the knee position based on ground surface conditions and facilitates maintaining the necessary maximum knee flexion to recover vertical postural stability during the landing process (Wu et al., 2013). In addition, when compared to the contributions of the ankle and hip joints during the landing, the knee joint’s eccentric control generates the majority of negative work in the sagittal plane. This is achieved by manipulating the knee flexion angular displacement to effectively absorb the impact from the ground (Augustsson et al., 2006; Tamura et al., 2021).

We observed that this knee extensor eccentric control mechanism is equally applied to StandTS movement. Similar to other landing or stair descent movements, knee extensor eccentric control plays a pivotal role in controlling falling momentum during the descending phase of StandTS. In this study, this “controlled” descending movement was well observed during the timeframe of StandTS, spanning from the onset of StandTS to the instant of initiation of vertical GRF descent (Figures 1, 3, from time point A to time point B). The instant of initiation of vertical GRF descent indicates the onset of rapid CoM vertical dropping. From the beginning of StandTS up to this point, knee extensor eccentric activation continually increased, reaching its peak at the initiation of vertical GRF descent (Figure 3, time point B). After this point, knee extensor eccentric activation sharply decreased, coinciding with a significant increase in CoM vertical descending velocity.

**Figure 6:**
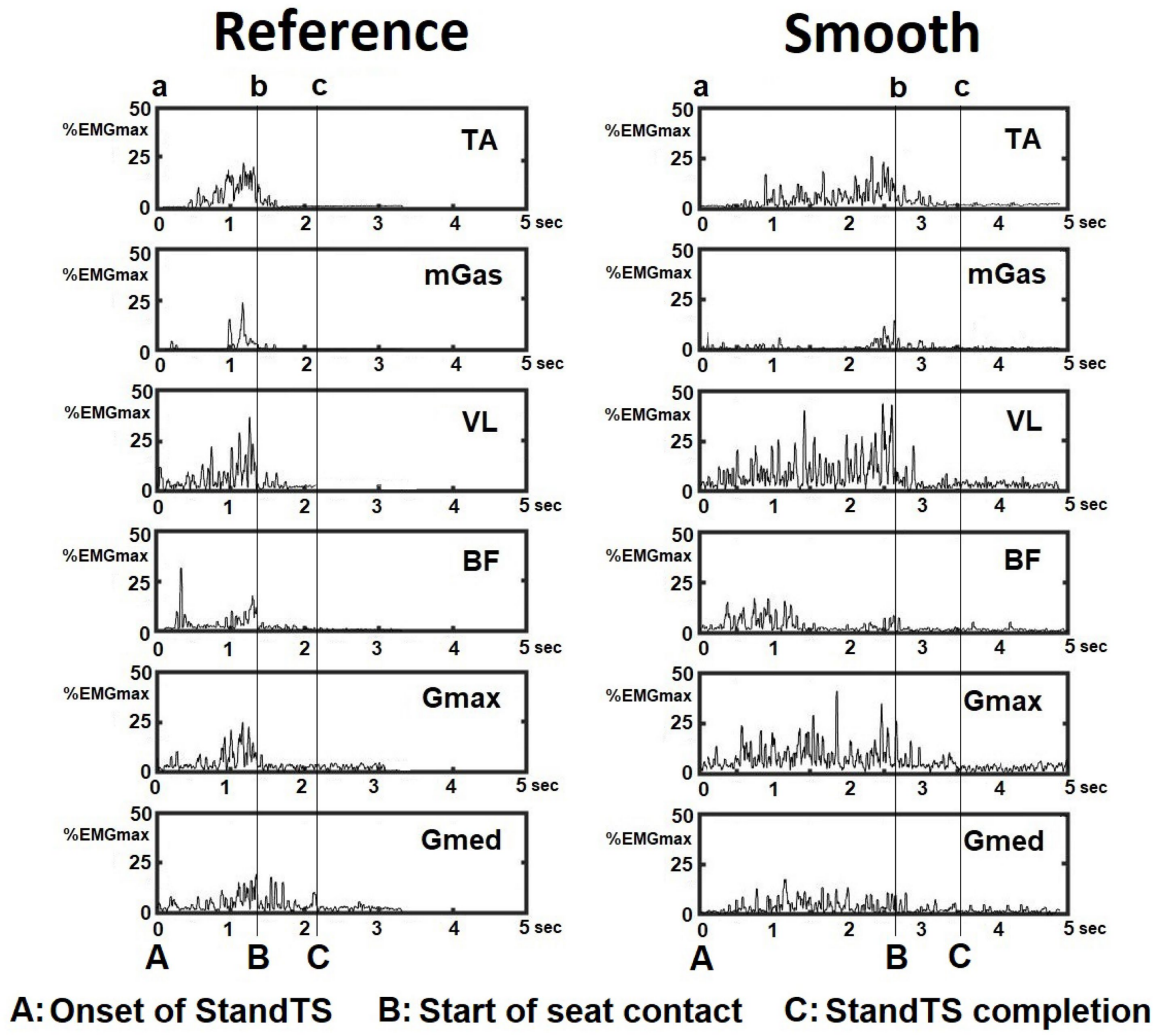
EMG activation pattern of leg muscles during the reference and smooth StandTS from a representative subject. Tibialis anterior (TA), medial gastrocnemius (mGas), vastus lateralis (VL), biceps femoris (BF), gluteus maximus (Gmax), and gluteus medius (Gmed).

Following the onset of StandTS, TA and Gmax activation commenced with ankle dorsiflexion and hip flexion to position the CoM of the body inside of the base of support in preparation for the descending phase. Upon the initiation of StandTS descent, TA and Gmax collaborated with VL, the knee extensor, to control the falling momentum of the CoM of the body during StandTS (Figure 6).

Notably, during the descending phase of StandTS, the distinctive feature of the knee extensor eccentric control mechanism was observed in its continuous activation increase from the onset of StandTS until right before the CoM vertical dropping (vertical GRF descent) (Figure 3, from time point A to time point B, quadriceps muscle (VL) activity). This highlights that the knee extensor remains activated until the last minute of the “controlled” descending phase before the leg muscle activation is released for the final accelerated descent for sitting.

The results of muscle synergy support these findings. In the reference-specific muscle synergy, the spatial structure results revealed an overwhelmingly high relative contribution of knee extensor eccentric control, accounting for almost 80%, compared to the contribution of the muscles at the ankle joint, with TA at approximately 30%, and the hip joint, with Gmax and Gmed at approximately 10% each (Figures 5B). The temporal activation of reference-specific muscle synergy demonstrated that this activation is maintained at a higher level during the descending phase of StandTS, emphasizing its crucial role in controlling falling momentum for postural stability during the act of sitting down.

In addition, our findings demonstrated a negative correlation between knee extensor eccentric work and postural sway (SDCoMaccel) in the vertical direction (Figure 4D, left panel). In other words, in both reference and smooth conditions, having better eccentric control at the knee joint during StandTS correlates with improved balance control in the vertical direction. Without sufficient knee extensor eccentric control, the body’s falling momentum is not adequately decelerated, increasing the potential risk of falls.

Previous studies have noted that, during the single-limb support (SLS) phase, knee extensor eccentric control of the perturbed leg served as the initial defense against potential falls following encountering ground surface challenges such as unexpected surface drop, slip, and trip (Pai et al., 2006; Wang et al., 2019; Jeon et al., 2022). This is due to the direct correlation between the level of knee extensor eccentric control and the risk of limb collapse, which, in turn, is closely associated with falls (Jeon et al., 2023b). Even in cases where limb collapse is avoided in the vertical direction, a weakened, wobbly, and unstable perturbed leg during SLS can still potentially lead to irrecoverable instability in the both A-P and M-L directions, and this exacerbates the instability of the compensatory balance recovery reactions, such as subsequent stepping and arm movements.

The function of this knee extensor eccentric control mechanism is equally applicable to maintaining postural stability during StandTS. Our findings showed that the smooth StandTS condition exhibited a greater knee flexion angular displacement along with increased knee joint work than the reference StandTS. Considering that the smooth StandTS condition demonstrated significantly less postural sway than the reference StandTS, this supports the notion that postural stability during the StandTS descending phase is primarily associated with achieving successful knee extensor eccentric control to decelerate the falling momentum of the body against gravity. Additionally, a good level of knee extensor eccentric control at the knee joint appeared to serve as a baseline or starting point to ensure effective balance control at other joints, such as the ankle, hip, and trunk, because it contributes to the overall efficiency of joint function for maintaining postural stability during StandTS.

### Increased contribution of the hip joint during smooth StandTS

Considering our aforementioned findings, it is reasonable to expect that the smooth StandTS condition would require greater knee extensor eccentric control at the knee joint than the reference StandTS condition. Indeed, the “controlled” eccentric knee flexion angular displacement and knee joint power are significantly increased during smooth StandTS compared to reference StandTS. However, a noteworthy aspect of the changes in leg muscle activation patterns observed in the smooth StandTS condition is the distinct enhancement in the contribution of the hip joint. Specifically, the spatial structure of smooth-specific muscle synergy showed that the relative contribution of Gmax and Gmed in the smooth StandTS was nearly twice as high as in the common condition (see Figure 5).

It is a well-known fact that the ability to swiftly control trunk motion consistently distinguishes older adults who may be at a heightened risk of falls (Grabiner et al., 2008), and hip extensor eccentric control plays an important role in this trunk movement control (Tateuchi et al., 2012). For example, the eccentric muscle activation of hip joint extensors serves to decelerate the CoM of the body in the A-P direction, counteracting external trunk flexion torques during dynamic movements such as walking and running (Teng and Powers, 2016).

The common muscle synergy results indicated that the majority of StandTS movement is primarily completed by muscle activation at the ankle and knee joints (Figure 5A). The reference-specific StandTS muscle synergy (Figure 5B) highlights that the contribution of the vastus lateralis (VL), representing the eccentric control of the knee extensor, is significantly greater than the contribution of muscles at other joints. However, in the smooth StandTS condition, due to the extended time required for controlling trunk flexion while maintaining StandTS descending balance, we observed an increase in the involvement of hip joint extensor (Gmax) and abductor (Gmed) muscles. The smooth-specific muscle synergy showed that the increase in hip extensor accounted for up to approximately 40% of the spatial structure of the muscle synergy (Figure 5C). Interestingly, this heightened hip joint involvement led to a notable decrease in the relative contribution of knee extensors (VL) compared to the reference-specific muscle synergy.

This type of kinetic relationship between knee and hip extensors has been previously observed during gait and running. For example, increased hip extensor eccentric work during hip flexion to decelerate the CoM of the body during walking and running results in reduced knee extensor eccentric work (Sasaki and Neptune, 2010). This shift allows for reduced reliance on knee extensors and increased dependence on hip extensors, contributing to efficient joint loading for body support (Sasaki and Neptune, 2010; Teng and Powers, 2016). This strategy, in turn, appeared to be similarly applied to the balanced StandTS movement. By increasing the contribution of hip extensor involvement during the smooth StandTS condition, the displacement of the body’s CoM in the A-P and vertical directions during the descending phase of StandTS can be controlled without excessive reliance on the knee extensors. This appears to be an important factor in diminishing postural sway in all directions during the smooth StandTS condition.

In addition, we found that M-L postural sway was negatively correlated to hip extensor work during the descending phase of StandTS. The preservation of M-L balance during StandTS is regarded as a more critical factor in maintaining postural stability for successful movement when compared to M-L balance control during the ascending phase of STS. Indeed, in the M-L direction, older fallers showed a significantly greater range of CoM displacement during the StandTS movement when compared to the CoM shift observed during the STS movement (Lin and Lee, 2022). Our findings demonstrated that the contribution of hip joint work is correlated with reduced postural sway in the M-L direction. Although hip joint work is not directly involved in ground impact absorption, which mainly occurs at the ankle and knee joint, positive work at the hip joint can contribute to additional StandTS balance control by facilitating energy transfer to other joints during the descending phase (Nagano et al., 2015). Such a contribution assists in effectively managing the falling momentum during StandTS.

### Clinical reflection

STS and StandTS movements are commonly used in clinical settings to evaluate mobility and assess fall risk in older adults (Tiedemann et al., 2008; Jeon et al., 2021b). These tests typically examine the number of STS completions within 30 s or the time taken to complete five STS, with no emphasis on the StandTS movement (Goldberg et al., 2012; McAllister and Palombaro, 2020). Even in balance tests specifically focusing on the StandTS movement, these tests rely on subjective assessments by testers, based on whether individuals use their hands during StandTS (e.g., as observed in tests such as the Berg Balance Scale and the Tinetti Performance Oriented Mobility Assessment).

Although StandTS may initially appear as a reverse movement of STS, a crucial distinction emerges as STS necessitates concentric knee extensor power, whereas StandTS relies significantly on knee extensor eccentric control to manage falling momentum against gravity. Notably, the effectiveness of eccentric control at the knee joint during the descending phase greatly influences potential fall risk (Wang et al., 2019; Jeon et al., 2022, 2023b,c). Our findings also highlighted the pivotal role of knee extensor eccentric control in descending balance control during StandTS, and work at the knee joint is correlated with reduced vertical postural sway. This underscores the importance of not overlooking the measurement of “balance control” ability, particularly associated with fall risk during the descending phase of sitting down, where the knee extensor eccentric control is crucial for maintaining postural stability. In addition, our findings demonstrate that smooth StandTS enables the prominent contributions of hip extensor and abductor muscles, leading to a more even distribution of weight-loading workload between the knee and hip joints, as well as improving M-L balance. These changes in balance control mechanisms induced by smooth StandTS motion provide direction for optimizing StandTS rehabilitation training to align with specific objectives.

Certainly, in the rehabilitation or clinical setting, there is a need to further elaborate on the assessment of postural stability to effectively classify the level of eccentric control during StandTS movement. For example, participants demonstrating the ability to smoothly lower themselves to a seated position by bending their knees in one continuous motion for a count of six metronome beats, as in smooth StandTS, would be considered as having great descending balance control during StandTS. The assessment of this smooth StandTS ability will provide valuable guidance for creating a more objective and accurate StandTS clinical test. In addition, there is an element of “movement awareness” that could be incorporated during smooth StandTS, which, if practiced regularly, may result in increased balance and balance confidence in patients with neurological disorders such as multiple sclerosis (Stephens et al., 2001).

### Limitation and future direction

Applying research findings from our study with healthy subjects to patient groups requires careful consideration and adaptation. This is because healthy individuals may not have the same underlying conditions, including factors such as muscle strength, joint stability, and pain; therefore, adjustments may be necessary to account for these differences.

Given our findings that smooth StandTS facilitates increased hip joint engagement, aiding in decreasing weight loading at the knee joint in the vertical direction, initial rehabilitation sessions for patients with knee joint issues such as arthritis or after knee replacement surgery can benefit from incorporating smooth StandTS exercises. This approach allows for beginning with light resistance at the knee joint, thus preventing a disproportionately higher weight loading at the knee joint.

It is a well-known fact that in knee osteoarthritis (OA), individuals unconsciously shift weight away from the affected compartment of the knee joint, potentially increasing the load on the healthier lateral side compartments (Pan et al., 2023). This leads to joint stiffness and reduced range of motion of both the affected and unaffected knee joints. To address this limited knee joint mobility and to decrease stress on surrounding structures, smooth StandTS exercise, which targets increased hip joint involvement for descending balance control, can effectively reduce excessive loading at the knee joint and minimize joint stiffness in the affected leg during sitting-down movement. This helps accommodate for limited knee joint mobility and reduces overall stress on surrounding structures.

Additionally, as smooth StandTS improves M-L postural stability during the movement, integrating smooth StandTS into rehabilitation training can help prevent fall risk in older adults during functional activities that require descending balance control, such as stair descent, descending a curb, and walking on uneven surfaces. Similarly, individuals with severe knee extensor muscle atrophy, such as patients with sporadic inclusion body myositis, can benefit from the smooth StandTS approach when practicing sitting-down movements without the use of ambulatory devices or assistance from their caregiver.

## Data availability statement

The raw data supporting the conclusions of this article will be made available by the authors, without undue reservation.

## Ethics statement

The studies involving humans were approved by the University of Texas at Tyler IRB review board. The studies were conducted in accordance with the local legislation and institutional requirements. The participants provided their written informed consent to participate in this study.

## Author contributions

WJ: Conceptualization, Data curation, Formal analysis, Funding acquisition, Investigation, Methodology, Project administration, Resources, Software, Supervision, Validation, Visualization, Writing – original draft, Writing – review & editing. XD: Conceptualization, Formal analysis, Investigation, Methodology, Software, Validation, Writing – original draft, Writing – review & editing. AD: Conceptualization, Formal analysis, Investigation, Methodology, Resources, Validation, Writing – original draft, Writing – review & editing. C-HG: Conceptualization, Formal analysis, Investigation, Methodology, Resources, Validation, Writing – original draft, Writing – review & editing.
